# Effect of Titanium Addition on the Thermal Properties of Diamond/Cu-Ti Composites Fabricated by Pressureless Liquid-Phase Sintering Technique

**DOI:** 10.1155/2014/713537

**Published:** 2014-03-04

**Authors:** Chih-Yu Chung, Chao-Hung Chu, Mu-Tse Lee, Chun-Ming Lin, Su-Jien Lin

**Affiliations:** Department of Materials Science and Engineering, National Tsing Hua University, Hsinchu 30013, Taiwan

## Abstract

In this study, minor-addition elements such as Si, Co, Cr, W, Mo, and Ti were added to matrix to improve the wettability between the diamonds and Cu matrix. The pressureless liquid-phase sintering technique adopted in this study provides a low-cost method for producing diamond/Cu composites with high potential for industrial mass production. Thermal properties of the diamond/Cu-Ti composites fabricated by pressureless liquid-phase sintering at 1373 K with variation in Ti contents were thoroughly investigated. XRD and TEM analysis show that TiC layer formed in the interface between Cu and diamond. The composites exhibited thermal conductivity as high as 620 W/m*·*K for 50 vol% diamond/Cu-0.6  at % Ti composite with diamond particle size of 300 µm. This value comes up to 85% of the thermal conductivity calculated by the Hasselman and Johnson (H-J) theoretical analysis. Under these conditions, a suitable coefficient of thermal expansion of 6.9 ppm/K was obtained.

## 1. Introduction

Conventional thermal management materials with thermal conductivities (above 200 W/m*·*K) and coefficients of thermal expansion (4–8 ppm/K) were summarized by Zweben [1]. However, owing to the increase in device power and integration levels [2], the demand for novel thermal management materials with high thermal conductivities is on the rise.

Due to their excellent thermal properties, synthetic diamonds with high thermal conductivity (1200–2000 W/m*·*K) are the most attractive thermal spreading material. However, diamond films utilized to spread heat are too thin to spread efficiently and it is very difficult to form bulk synthetic diamond. Therefore, high thermal conductive metals, such as Al, Ag, and Cu, were utilized as binders to form bulk composites [3–7] reinforced with diamond to match this demand. However, due to the poor wettability between diamond and metal, the interfaces between diamond and metal are not in well contact and this will be detrimental for heat conduction between the interfaces. There are two alternative methods to improve the contact between diamond and metal. The first method is to fabricate composites under high pressure (above 1 GPa) and high temperature (above the melting point of metal) process conditions which requires expensive and complex equipment [8]. The second method can be achieved through carbide-forming elements addition to form a thin carbide layer at the interface and to improve the contact and wettability between diamond and metal [9–16].

In recent years, high thermal conductive diamond/metal composites used as heat sink have been fabricated by various techniques with high pressure, expensive, and complex equipment, such as hot pressing [17], gas pressure infiltration [18–20], and spark plasma sintering [21]. In this study, diamond/Cu composites were fabricated by pressureless liquid-phase sintering technique with minor-addition elements to improve the contact and wettability between the diamond and Cu matrix. The results in this study demonstrate that pressureless liquid-phase sintering technique is a very promising process for fabricating diamond/Cu-Ti composites with the potential for mass production.

## 2. Materials and Methods

Saw diamond powders with thermal conductivity of 1800 W/m*·*K and a particle size of 300 *μ*m (YK-9E, Fine Abrasives Taiwan Company) were used as reinforcements. Cu powders with thermal conductivity of 400 W/m*·*K and with a particle size of 5 *μ*m (purity in 99.9 wt%, Artc-1000 A, Advance Research Technology Corporation) and minor-addition element powders (Si, Co, Cr, W, Mo, and Ti) with a particle size of 100 *μ*m (purity in 99.9 wt%, Alfa Aesar) were mixed as matrix materials.

Diamond, copper, and minor-addition element powders were mechanically mixed and compacted under 700 MPa for 10 minutes to fabricate diamond/Cu composites with a diameter of 12.9 mm and height of 3 mm and then sintered in tube furnace with H_2_ (100 sccm) gas flow under vacuum (1.8 torr) at 1373 K for 30 minutes.

Micrographs and element compositions of diamond/Cu-Ti composites were analyzed by a scanning electron microscope (SEM, JEOL-5410) and an X-ray diffractometer (XRD, Rigaku_D/DMAXIIB). Thermal conductivity was measured via laser-flash analyzer (LFA, Netzsch-LFA 447). Coefficient of thermal expansion (CTE) was measured by thermal analysis apparatus (TMA, Seiko-SSC5200) with a heating rate of 3 K/min, ranging from 373 K to 473 K. The Archimedes method was applied to measure the densities of the composites and the relative densities were obtained by comparing measured densities with the theoretical densities. Focused ion beam (FIB, FEI-Nova 200) technology was used for the preparation of transmission electron microscope (TEM, JEOL-JEM3000F) samples. The samples were then observed under TEM to reveal the boundary regions of the diamond/Cu interface.

## 3. Results and Discussion

### 3.1. Diamond/Cu Composites with Varying Minor-Addition Elements

**Figure 1:**
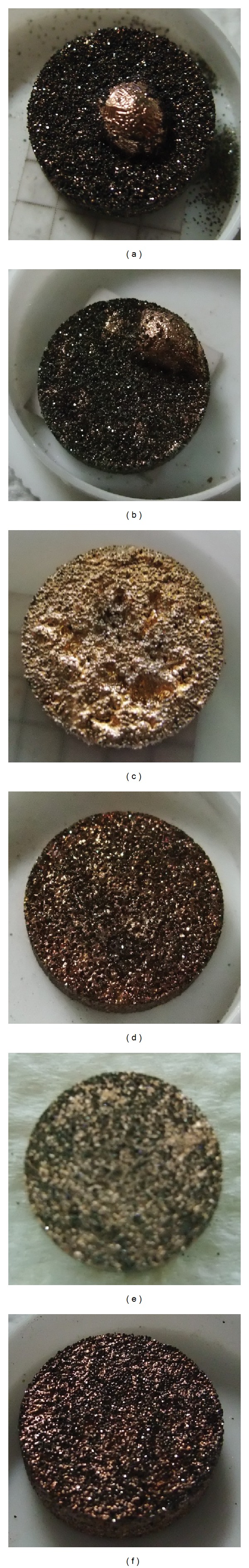
The sample images of 50 vol% diamond/Cu composites with 3 at % varying minor-elements addition: (a) Si, (b) Co, (c) Cr, (d) W, (e) Mo, and (f) Ti.

The sample images of 50 vol% diamond/Cu composites with 3 at % varying minor-addition elements are shown in Figure 1. It can be seen that only W, Mo, and Ti addition can form integral-shape composites without matrix repelling from the composites. Of all the chosen minor-addition elements, W, Mo, and Ti all belong to the carbide-forming elements. The other minor-addition elements added to the composites are incapable of forming integral-shape composites due to matrix repelling from the composites during the sintering process.

**Figure 2:**
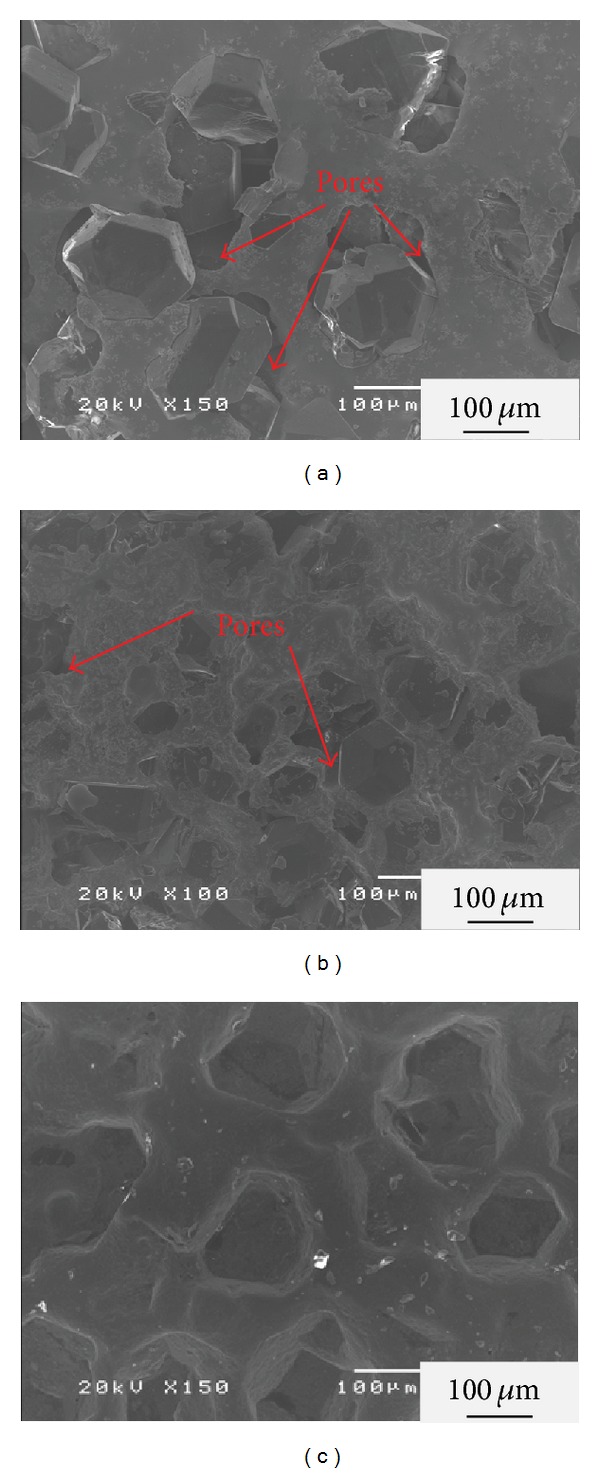
The surface SEM images of the 50 vol% diamond/Cu composites with (a) 3 at % W addition, (b) 3 at % Mo addition, and (c) 3 at % Ti addition.

Figure 2 shows the surface SEM images of the 50 vol% diamond/Cu composites with 3 at % (a) W, (b) Mo, and (c) Ti additions. Figures 2(a) and 2(b) show apparent interfacial pores at the boundaries between the diamonds and Cu matrix. It is considered that W and Mo additions can form integral-shape composites but cannot improve the contact between the diamonds and Cu matrix efficiently. In Figure 2(c), it can be seen that the diamonds and Cu matrix are in well contact at the boundaries and the Cu matrix can cover the diamond surfaces entirely. Furthermore, the thermal conductivities of the composites are 209, 257, and 348 W/m*·*K for 3 at % W, Mo, and Ti additions, respectively. The highest value of thermal conductivity was obtained with Ti addition in this study. Therefore, Ti is regarded as the most suitable carbide-forming element in this study to improve the contact and wettability at the boundaries between the diamonds and Cu matrix.

### 3.2. Microstructure and Composition Analysis of Diamond/Cu-Ti Composites

**Figure 3:**
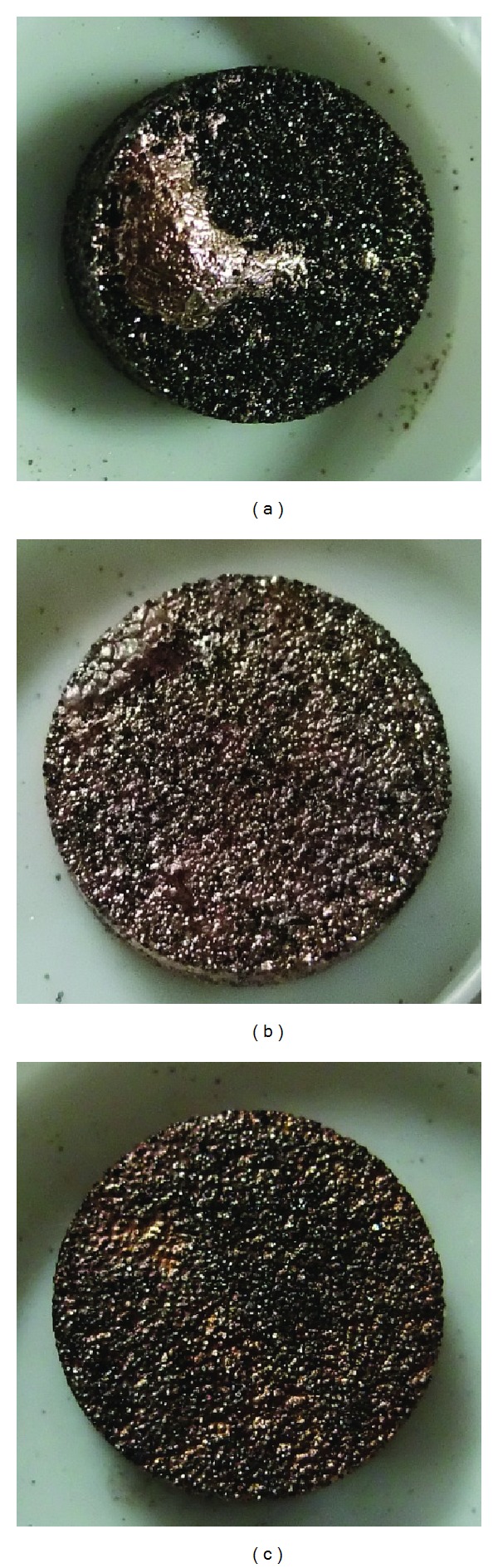
The sample images of the 50 vol% diamond composites with varying Ti addition in the Cu matrix: (a) 0.2 at % Ti, (b) 0.3 at % Ti, and (c) 0.6 at % Ti.

The sample images of the 50 vol% diamond composites with varying Ti addition (0.2, 0.3, and 0.6 at %) in the Cu matrix are shown in Figure 3. It can be seen that the addition of 0.2 at % Ti (Figure 3(a)) is not enough to form an integral-shape composite without matrix repelling. The integral-shape composites can be achieved only above 0.3 at % Ti addition. Composite with 0.6 at % Ti addition has a good integral-shape as shown in Figure 3(c).

**Figure 4:**
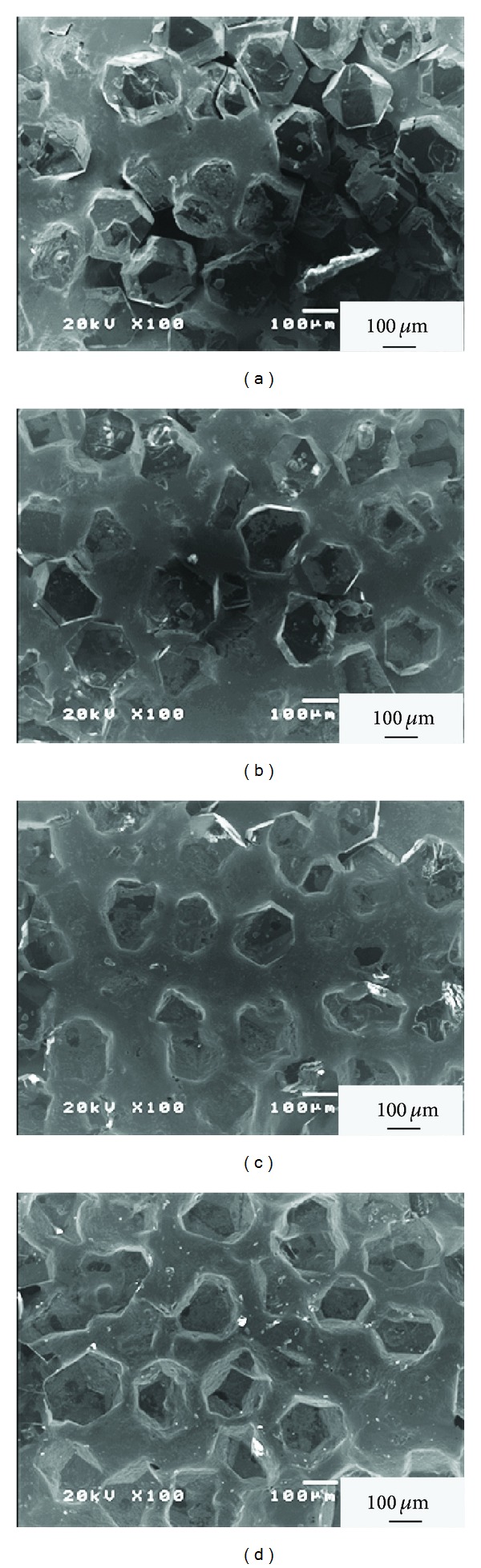
The surface SEM images of the 50 vol% diamond composites with varying Ti addition in the Cu matrix: (a) 0.3 at % Ti, (b) 0.4 at % Ti, (c) 0.6 at % Ti, and (d) 0.9 at % Ti.

Figure 4 shows the surface SEM images of the 50 vol% diamond composites with varying Ti addition in the Cu matrix.  Figure 4(a) shows apparent interfacial pores at the boundary between the diamonds and the matrix appearing in Cu-0.3 at % Ti composites. With increasing Ti content, fewer interfacial pores were found in the contact regions between the diamonds and the Cu matrix. When the Ti content increased to 0.6 and 0.9 a t%, there were no apparent interfacial pores and the diamond surface was entirely covered by the copper matrix as shown in Figures 4(c) and 4(d). These phenomena indicate that increasing Ti content improves the wettability and contact between Cu matrix and diamond surface effectively, and therefore less interfacial pores were produced during the process.

**Figure 5:**
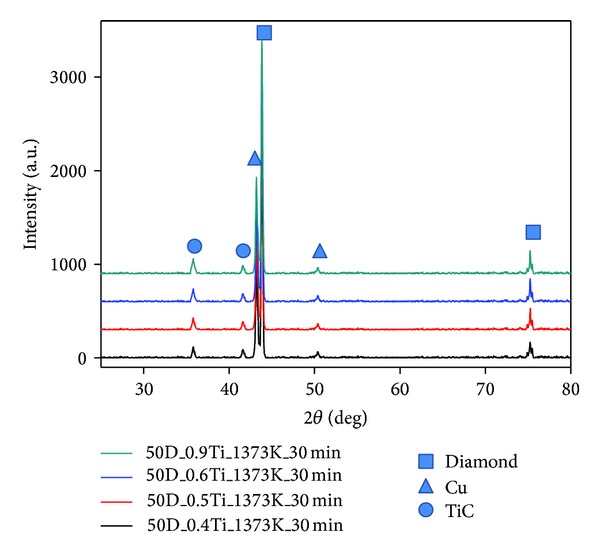
The X-ray diffraction patterns of the 50 vol% diamond/Cu-Ti composites with varying Ti content.

The X-ray diffraction patterns of the diamond/Cu-Ti composites, shown in Figure 5, indicate that the peaks only belonged to diamond, Cu, and TiC. It can be deduced that TiC forms during the sintering process. The peak intensity grows stronger with increasing Ti content, indicating greater amount of TiC formation.

**Figure 6:**
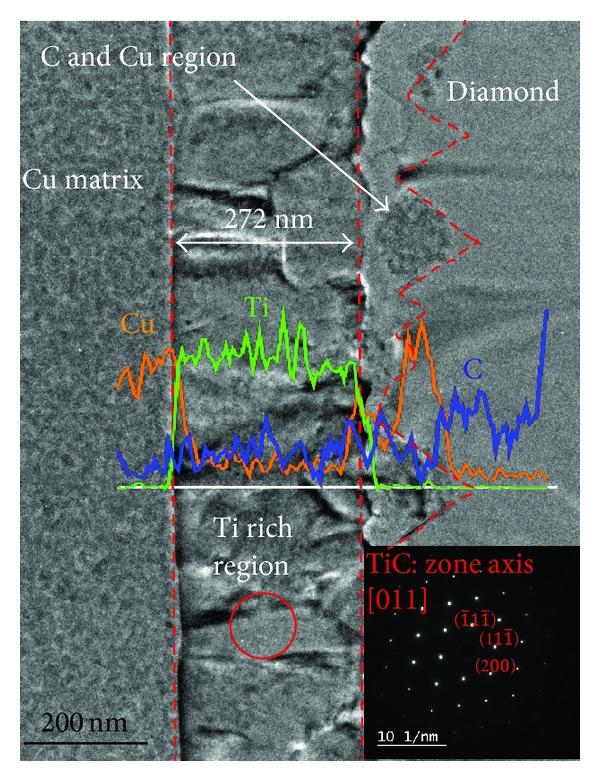
TEM bright field image and EDS interface line-scan signals of a 50 vol% diamond/Cu-0.6 at % Ti composite interface. The insert is the diffraction pattern of columnar structure (red circle region).

Figure 6 shows the TEM bright field image and EDS interface line-scan signals of a 50 vol% diamond/Cu-0.6 at % Ti composite interface. The interface between Cu matrix and diamond is composed of a columnar structure near Cu and an irregular layer near diamond. The C, Ti, and Cu EDS line scans were used to determine the distribution of element across the interface, as shown in Figure 6. It is clear that the scan path consists of four zones: (a) Cu matrix region, (b) Ti-rich columnar region, (c) Cu-C irregular region, and (d) diamond region. The insert in Figure 6 is the diffraction pattern of circle region in Ti-rich columnar structure and indicates that this columnar structure is a TiC crystal structure. This is consistent with the X-ray diffraction analysis as shown in Figure 5. This columnar TiC layer is about 270 nm in thickness. The Cu-C irregular layer is comprised of Cu and C which may have resulted from the reaction during sintering. The structure of this irregular layer is composed of Cu and C. However, the mechanism for the formation of this interfacial structure still requires further investigation.

### 3.3. Thermal Conductivity and Coefficient of Thermal Expansion (CTE)

**Figure 7:**
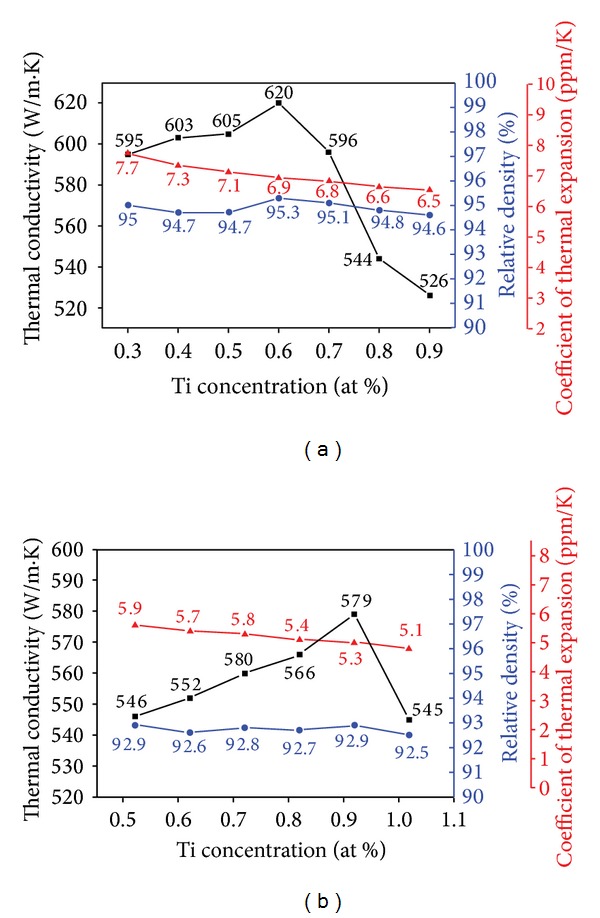
The curves of thermal conductivity, relative density, and coefficient of thermal expansion versus Ti content with (a) 50 vol% diamond and (b) 60 vol% diamond.

Figure 7 shows the curves of relative density, thermal conductivity, and coefficient of thermal expansion versus Ti content with (a) 50 vol% diamond and (b) 60 vol% diamond. It can be seen that the relative densities decrease with increasing diamond volume fraction, due to the increase in interfacial areas and the more bridging effect between diamonds.

Figure 7 indicates that initially the thermal conductivity values increase with increasing Ti addition, upon reaching a maximum, and then the thermal conductivities begin to show apparent decrease with further addition. The Ti addition enhanced the wettability between diamond and Cu, thus promoting the interfacial contact between the matrix and diamond during pressureless liquid-phase sintering and increasing the thermal conductivities of the composites. Thermal conductivity of the highest 620 W/m*·*K value can be achieved for 50 vol% diamond/Cu-0.6 at% Ti composite. However, upon exceeding 0.7 at% Ti addition, the thermal conductivity decreases drastically.

It is considered that varying amounts of diamond surface areas need varying Ti addition to improve the wettability of the interface and then have the highest thermal conductivity in each diamond volume fraction condition. By assuming diamonds as spheres with a diameter of 300 *μ*m for simplified calculation, the diamond surface areas of the 50 vol% condition (the volume of the composite is 3.92 × 10^−7^ m^3^) are 3.92 × 10^−3^ m^2^ and 60 vol% condition (the volume of the composite is 3.92 × 10^−7^ m^3^) is 4.7 × 10^−3^ m^2^. Considering that the highest thermal conductivity of 50 vol% condition is obtained with 0.6 at % Ti addition and comparing the surface areas values of 50 vol% and 60 vol%, it can be calculated that the optimum Ti addition in 60 vol% condition is 0.92 at%. The experimental results verify this calculation, as shown in Figure 7(b). The 60 vol% diamond composites exhibit the highest thermal conductivity up to 579 W/m*·*K with 0.92 at% Ti content. However, upon exceeding this optimum 0.92 at% Ti addition, the thermal conductivity decreases drastically as the trend in the 50 vol% diamond composites.

The above results indicate that suitable Ti addition and TiC thickness strengthen the interfacial bonding to improve thermal conductivity. However, the thermal conductivities of the composites later decreased owing to the excess formation of the TiC interphase (Figure 6) with low thermal conductivity during the sintering process. Thermal conductivities of 60 vol% diamond composites are lower than those of 50 vol% diamond composites. These can be attributed to lower relative densities of the composite under 60 vol% conditions. It is considered that the suitable Ti addition in each diamond volume fraction condition is proportional to the amounts of diamond surface areas.

The CTE values in this study are in the range of 5 to 8 ppm/K, which are the adequate values for thermal management materials that are to be utilized in electronic devices [1]. The CTE values of the composites decrease monotonously from 7.7 to 6.5 ppm/K (for composites with a diamond content of 50 vol% and 0.3–0.9 at % Ti) or from 5.9 to 5.1 ppm/K (for composites with a diamond content of 60 vol% and 0.52–1.02 at% Ti) with increasing Ti content. This can be explained by the improvement in bonding at the interface with higher content of Ti addition. The CTE values of 60 vol% diamond composites are lower than those of 50 vol%. This is due to the fact that the low CTE value of diamonds (1.3 ppm/K) provides greater contribution to the CTE values of composites comprised of higher diamond content, thus reducing the final CTE value outcomes of the composites.

In recent years, high thermal conductive Cu/diamond composites with thermal conductivities of 700, 657, 493, and 615 W/m*·*K fabricated by high-cost techniques have been published. The composites produced in this study demonstrate thermal conductivities comparable with findings in previous literature made by other authors [18, 21–23]. However, in comparison with these techniques, the fabrication technique applied in this study provides a low-cost, simple alternative to fabricate diamond/Cu composites with great potential for industrial mass production.

### 3.4. Theoretical Analysis of Thermal Conductivity

In order to fully understand the thermal conduction behavior in diamond/Cu-Ti composites, it is crucial to compare the experimental results with theoretical predictions. The Hasselman and Johnson (H-J) model applied in combination with effective medium approximation (EMA) scheme is the most popular predictive method, in which the Kapitza resistance effect and particle size are taken into consideration [23]:

(1) $$\begin{array}{c} K_{c}=\frac{K_{m}\left[2K_{m}+K_{p}^{\text{eff}}+2\left(K_{p}^{\text{eff}}-K_{m}\right)V_{p}\right]}{2K_{m}+K_{p}^{\text{eff}}-\left(K_{p}^{\text{eff}}-K_{m}\right)V_{p}}, \end{array}$$

where *K* is the thermal conductivity, *V* is the volume fraction of reinforcement, and the subscripts *c*, *m*, and *p* refer to the composite, matrix, and reinforcement particles, respectively. The effective thermal conductivity of reinforcement particles, *K*_*p*_^eff^, is defined as [23]:

(2) $$\begin{array}{c} K_{p}^{\text{eff}}=\frac{K_{p}}{1+2R_{k}K_{p}/d}, \end{array}$$

where *d* is the average diameter of reinforcements and *R*_*k*_ is the interfacial resistance (Kapitza resistance). However, the model was made under the assumption that the interface between the matrix and reinforcements is perfect and sharp [23, 24]. In the case of this study, interphases formed at the interface, which contributed additional thermal resistance to the initially assumed diamond/metal sharp interface. To obtain an accurate model for thermal conductivity prediction, the thermal resistance of the interphases should be considered. In order to simplify the calculation, the Cu-Ti-C irregular layer is still neglected. The interfacial thermal resistance can be expressed by

(3) $$\begin{array}{c} R_{k}=R_{\text{Cu}/\text{TiC}}+R_{\text{TiC}}+R_{\text{TiC}/\text{Diamond},}\\ R_{\text{TiC}}=\frac{\text{thickness}\;\text{of}\;\text{TiC}}{K_{\text{TiC}}}, \end{array}$$

where *R*_Cu/TiC_, *R*_TiC_, and *R*_TiC/Diamond_ are the thermal resistance of Cu/TiC interface, TiC interphase, and TiC/Diamond interface, respectively, and *K*_TiC_ is the thermal conductivity of TiC.

The thermal resistance, *R*_Cu/TiC_ and *R*_TiC/Diamond_, can be described by diffuse mismatch model (DMM). This interphase thermal resistance, *R*_*i*/3-*i*_, based on DMM model is related to the phonon velocities (*v*) in the *i* phase and the 3-*i* phase, as well as the absolute temperature *T*, can be expressed in the following form [23]:

(4) $$\begin{array}{c} R_{i/3\text{-}i}={\left[1.02\times 10^{10}\frac{\left(\sum_{j}v_{i,j}^{-2}\right)\left(\sum_{j}v_{3\text{-}i,j}^{-2}\right)}{\sum_{i,j}v_{i,j}^{-2}}\right]}^{-1}\times T^{-3}, \end{array}$$

where the subscripts *i* and 3-*i* refer to the two adjacent phases on each side of the interface. The subscript *j* represents the mode of phonon velocity (longitudinal or transverse).

In this modeling, the effect of porosity was taken into account by reassigning an effective thermal conductivity to the composite. The effective thermal conductivity of the composite was derived from combining the thermal conductivity of composite and air according to the rule of mixture (ROM):

(5) $$\begin{array}{c} K_{c\text{-}p}=K_{c}\times V_{c}+K_{\text{air}}\times V_{\text{porosity}}, \end{array}$$

where *K*_*c*-*p*_ represents the thermal conductivity of composites containing pores. *K*_*c*_ is the result of thermal conductivity of the composite calculated by (1)–(4). *V*_*c*_ and *V*_porosity_ are the volume fraction of composite and porosity, respectively.

Table 1 shows the longitudinal, transverse phonon velocities [24, 25] and thermal conductivities of Cu, TiC, and diamond in theoretical values. Combining the above equations (1)–(5) and theoretical values, the values of the interfacial resistance and theoretical thermal conductivity can be calculated as shown in Table 2.

The calculated theoretical thermal conductivity is 725 W/m*·*K and the experimental value is 620 W/m*·*K as shown in Table 2 and Figure 7(a), respectively. It appears that the experimental value comes up to 85% of the theoretical value. The good comparison results between the theoretical analysis and experimental measurements support the feasibility of incorporating the consideration of additional interfacial thermal resistance effect caused by the interphase into H-J model.

## 4. Conclusions

Diamond/Cu-Ti composites with high thermal conductivities can be successfully fabricated via pressureless liquid-phase sintering method. XRD and TEM analysis show that the TiC layer formed at the interface between Cu and diamond. Under the condition of 50 vol% diamond/Cu-0.6 at % Ti, the composite shows the highest value of thermal conductivity of 620 W/m*·*K and a low CTE value of 6.9 ppm/K, which comes up to 85% of the thermal conductivity calculated by the Hasselman and Johnson (H-J) theoretical analysis. Applying pressureless liquid-phase sintering method to fabricate high thermal conductive composites provides an easy and cost-effective alternative method to produce diamond/Cu-Ti composites with high thermal conductivities for thermal management applications.

## Figures and Tables

**Table 1:** The longitudinal, transverse phonon velocity and thermal conductivity of Cu, TiC, and diamond in theoretical calculation [24, 25].

Materials	Phonon velocity *v*_longitudinal_ (×10^3^ m/s)	Phonon velocity *v*_transverse_ (×10^3^ m/s)	Thermal conductivity *K* (W/m·K)
Cu	4.8	2.3	380
TiC	10.1	7.3	19
Diamond	17.5	12.8	1800

**Table 2:** The calculation values of the interfacial resistance and theoretical thermal conductivity of the 50 vol% diamond/Cu-0.6 at % Ti composite.

*R*_TiC/diamond_ (m^2^·K/W)	*R*_TiC_ (m^2^·K/W)	*R*_Cu/TiC_ (m^2^·K/W)	*R*_*k*_ (m^2^·K/W)	*K*_*p*_^eff^ (W/m·K)	*K*_*c*_ (W/m·K)	*K*_*c*-*p*_ (W/m·K)
5.2 × 10^−10^	1.4 × 10^−8^	1.4 × 10^−10^	1.5 × 10^−8^	1530	761	725
